# Prevalence and risk factors of anxious and depressive symptoms in first-trimester females and their partners: a study during the pandemic era of COVID-19 in China

**DOI:** 10.1186/s12888-023-04621-2

**Published:** 2023-03-03

**Authors:** Xuemei Qin, Weiling Zhang, Shuyin Xu, Mohan Ma, Xing Fan, Xueqing Nie, Jin Liu, Yumeng Ju, Li Zhang, Lingjiang Li, Yan Zhang, Bangshan Liu

**Affiliations:** 1 Department of Psychiatry, and National Clinical Research Center for Mental Disorders, The Second Xiangya Hospital of Central South University, Changsha, Hunan 410011 China; 2 China National Technology Institute on Mental Disorders, Hunan Technology Institute of Psychiatry, Hunan Key Laboratory of Psychiatry and Mental Health, Hunan Medical Center for Mental Health, Mental Health Institute of Central South University, Changsha, Hunan 410011 China; 3 Changsha Hospital for Maternal & Child Health Care, Changsha, Hunan 410007 China

**Keywords:** Anxiety, Depression, Family functioning, Quality of life, Early pregnancy

## Abstract

**Background:**

The pandemic of coronavirus disease 2019 lastingly affects public mental health. Many studies have described symptoms of anxiety and depression in pregnant women before the pandemic. However, the limited study focuses on the prevalence and risk factors of mood symptoms among first-trimester females and their partners during the pandemic in China, which was the aim of the study.

**Methods:**

One hundred and sixty-nine first-trimester couples were enrolled. The Edinburgh Postnatal Depression Scale, Patient Health Questionnaire-9, Generalized Anxiety Disorder 7-Item, Family Assessment Device-General Functioning (FAD-GF), and Quality of Life Enjoyment and Satisfaction Questionnaire, Short Form (Q-LES-Q-SF) were applied. Data were mainly analyzed through logistic regression analysis.

**Results:**

17.75% and 5.92% of first-trimester females had depressive and anxious symptoms, respectively. Among partners, 11.83% and 9.47% had depressive and anxious symptoms, respectively. In females, higher scores of FAD-GF (OR = 5.46 and 13.09; *P* < 0.05) and lower scores of Q-LES-Q-SF (OR = 0.83 and 0.70; *P* < 0.01) were related to the risk of depressive and anxious symptoms. Higher scores of FAD-GF were associated with the risk of depressive and anxious symptoms in partners (OR = 3.95 and 6.89; *P* < 0.05). A history of smoking was also related to males’ depressive symptoms (OR = 4.49; *P* < 0.05).

**Conclusion:**

This study prompted prominent mood symptoms during the pandemic. Family functioning, quality of life, and smoking history increased risks of mood symptoms among early pregnant families, which facilitated the updating of medical intervention. However, the current study did not explore interventions based on these findings.

## Introduction

Perinatal depressive and anxious symptoms are common in low- and middle-income countries and affect about 20% of females [1]. They not only increase the risk of spontaneous abortion, preeclampsia [2], and postpartum psychiatric disorders [3] in pregnant females but also affect the growth of the offspring’s emotions, socio-environmental development, and partner’s mental health status [4]. The coronavirus disease 2019 (COVID-19) pandemic has significantly influenced public mental health [5] and pregnant females are particularly vulnerable to engaging in emotional problems during this pandemic. A study in Qatar reported a high prevalence of anxiety (34.4%) and depression (39.2%) symptomatology among females during the whole pregnancy period [6]. Another study in China indicated that the prevalence of anxious and depressive symptoms reached 13.4% and 35.4% in the third trimester, respectively [7].

The first trimester of pregnancy is the period before the end of the 13th week of gestation [8]. Research during the pandemic in China emphasized that the first trimester of pregnancy was associated with an increased risk of anxious and depressive symptoms in pregnant females [9], consistent with studies before the pandemic indicating that depression and anxiety levels in early pregnancy were higher compared to other trimesters [10]. However, the limited studies have reported on the prevalence of anxious and depressive symptoms in first-trimester pregnant females, especially during the pandemic in China.

Meanwhile, various studies have investigated risk factors of prenatal anxious and depressive symptoms before the pandemic. A history of alcohol and smoking would escalate the risk of early prenatal anxiety [11, 12]. Sociodemographic variables, including marital status and smoking history [11], and a history of depressive disorder [13] can increase the likelihood of early prenatal depressive symptoms. Childhood traumatic events were reported to predict depressive symptoms during pregnancy as well [14]. However, understanding risk factors for depressive and anxious symptoms in first-trimester women during the pandemic of COVID-19 in China remains ambiguous.

Furthermore, the mental health of women’s partners during pregnancy is also of concern. As studies suggested, expectant fathers are prone to psychological distress and anxiety [15]. About 11% of them had symptoms of depression and anxiety [16], which is very likely to negatively impact the fetus, pregnant women, and themselves [17]. The pandemic era has affected the economy, and increased employment and financial stress [18], which might cause significant stress among males [19]. However, few studies have examined the prevalence of anxious and depressive symptoms in male partners during early pregnancy under the context of the pandemic, which helps to clarify the impact of the outbreak on partners. Furthermore, although factors such as low self-esteem and dissatisfaction with the marital relationship have been reported to be associated with the partners’ depressive and anxious symptoms [20], research on risk factors for their mood symptoms in early pregnancy remains inadequate, especially during the pandemic.

Therefore, the present study aimed to investigate the prevalence and risk factors of anxious and depressive symptoms among first-trimester females and their partners during the COVID-19 pandemic era in China, which might facilitate proactive, multidisciplinary, and integrated health intervention for them.

## Methods

### Data source

This cross-sectional study was performed in the outpatient clinic of Obstetrics and Gynecology at Changsha Hospital for Maternal & Child Health Care from December 2020 to July 2021 based on convenience sampling. Each woman in early pregnancy at her initial prenatal visit was introduced to the purpose and content of this study by a researcher. The one who was willing to participate would be given a self-reported assessment booklet to complete with the assistance of a researcher. Meanwhile, the partners of pregnant women enrolled in the present study would also be asked if they would like to participate. To save their time in the clinic, male partners willing to participate were provided a Quick Response code to access the online survey system via WeChat to complete the assessments. Therefore, there were 283 pregnant women agreed to participate while only 197 partners. In total, 169 paired couples were enrolled based on the following inclusion-exclusion criteria. Each participant signed an informed consent form, and anonymity was guaranteed. This study followed ethical guidelines and received ethical approval.

### Inclusion-exclusion criteria

To be eligible for inclusion, the participants need to be: (1) ethnic Han; (2) right-handed; (3) junior high school or higher education; (4) currently living with a partner; (5) willing to participate in this research. Specifically, for pregnant females, the study only included participants aged from 18 to 40 who were pregnant within 13^+ 6^ weeks.

To control participants who present with additional characteristics that could interfere with the study’s validity, we excluded participants with the following characteristics: (1) pre-existing mental disorder that is other than a depressive disorder or anxiety disorder (such as schizophrenia, schizoaffective disorder, bipolar disorder, etc.); (2) history of alcohol or other psychoactive substance dependence; (3) history of organic brain disease or other serious physical illness. For pregnant females, we also excluded people with serious infections, trauma, immune disorders, or other major medical illnesses during pregnancy (e.g., Cushing’s disease, thyroid disease, gestational hypertension, gestational diabetes mellitus, etc.).

### Assessments

#### General information

The self-reliant general social demographic and clinical information were applied to participants. The height and weight of the participants were measured in the outpatient clinic. The BMI values were derived by computer and applied to the statistical analysis to explore whether it is a risk factor for mood symptoms based on prior studies [21]. Drinking history was defined as at least one alcoholic beverage per month, three months before pregnancy on average. Smoking history was defined as smoking tobacco products at least once a day for a month or longer before pregnancy.

#### Depressive and anxious symptoms

1) Edinburgh Postnatal Depression Scale (EPDS) was used to evaluate pregnant females’ depressive symptoms [22]. It consists of 10 items, and each is graded by a four-point Likert scale, from never (zero points) to always (three points), with items 1 and 2 being reversed. The total score ranges from 0 to 30. In this study, an EPDS score ≥ 10 was defined as having significant depressive symptoms with reference to previous studies [1, 23]. The Chinese version of EPDS has satisfactory psychometric properties in detecting depression in Chinese women during pregnancy [24]. Cronbach’s α of the EPDS was 0.83 in this study.

2) Patient Health Questionnaire-9 (PHQ-9) was used to evaluate the partner’s depressive symptoms, which consists of 9 items [25]. The questionnaire scores each of the nine DSM-5 criteria with a four-point Likert scale, from never (zero point) to almost every day (three points). The reliability and validity of the Chinese version of PHQ-9 for screening depression have been verified [26]. In this study, Cronbach’s α of the PHQ-9 was 0.87 and a score of PHQ-9 ≥ 10 was defined as significant depressive symptoms [27].

3) Generalized Anxiety Disorder-7 (GAD-7) is a screener for generalized anxiety disorder [28] and was performed on both pregnant females and their partners. It consists of 7 items, and each is graded on a four-point Likert scale with a total score ranging from 0 to 21. This scale offers an acceptable psychometric performance in screening prenatal anxiety in mainland China [29] and has been used on Chinese pregnant women during the epidemic of COVID-19 [30]. In this study, Cronbach’s α of the GAD-7 was 0.87 in pregnant females and 0.92 in partners, and GAD-7 ≥ 10 was considered a clinically significant moderate to severe anxious symptom, which was based on the original study and previous researches in the perinatal period [31–34].

#### Psychosocial factors

1) Family Assessment Device-General Functioning (FAD-GF) is a subscale of FAD with 12 items to evaluate family functioning [35] in participants of the current study. Each item scores on a four-point Likert scale from (one point) very likely to (four points) very unlikely. The average score on 12 items is the final score, and the higher the score, the worse the family function. Both the Chinese version of FAD and the GF subscale possess good psychometrics in Chinese samples [36, 37]. In the current study, Cronbach’s α of the FAD-GF was 0.87 in females and 0.80 in males.

2) Quality of Life Enjoyment and Satisfaction Questionnaire, Short Form (Q-LES-Q-SF), Childhood Trauma Questionnaire-28 Item Short Form (CTQ-SF), and Social Support Rating Scale (SSRS) were only used for pregnant women. The Q-LES-Q-SF assessed the quality of life enjoyment and satisfaction with 16 items [38] and has adequate validity and reliability in the Chinese population [39]. In the present study, Cronbach’s α of the Q-LES-Q-SF was 0.89. Each item of this scale scores on a five-point Likert scale in satisfaction level from very unsatisfied (one point) to very satisfied (five points). The final grade will be the sum of 14 items in total.

3) The CTQ-SF was included to assess childhood traumatic experiences [40] and consists of 28 items, each of which is graded from never (zero point) to often (four points). This scale has good reliability and validity in Chinese depressive samples [41] and its Cronbach’s α in the current study was 0.76.

4) Finally, the SSRS is a self-report measure of enacted social support [42] shown excellent validity and reliability, and has been widely used for perinatal women in China [43, 44]. The Cronbach’s α of SSRS in this study was 0.65. This scale consists of 10 items and can be divided into three dimensions (subjective social support, object social support, and the utilization of support). The total scores of SSRS range from 0 to 40 with scores < 20 indicating limited social support, 20–30 representing the general level of social support, and 30–40 representing satisfying social support.

### Statistical analysis

All statistical analyses were performed on IBM SPSS for Windows, version 20.0 (IBM Corp., Armonk, NY, USA). Data were presented as mean (standard deviation), median (interquartile range, IQR), or n (%) in different categories. Spearman correlation and partial correlation analysis were used to investigate factors correlated with anxious and depressive symptoms. A two-tailed test was set in the GPower 3.1 software with a test level *α* of 0.05 and an effect size of 0.3 (medium) for post hoc analysis, and the test power (1-*β*) for this study was calculated to be 0.98. Variables significant in correlation analyses, and further, with *P* ≤ 0.1 in the univariate logistic regression analysis were entered as covariates in the binomial multivariate logistic regression analysis. Odds Ratios (OR) and 95% Confidence Intervals (CI) were used to estimate the effects of risk factors on anxious and depressive symptoms. Statistical significance is defined as a two-tailed *P* < 0.05.

## Results

### Characteristics of females and their partners

In this study, the mean gestational days of pregnant females was about 48 days (48.11 ± 12.03). Female participants were about 29 years old (29.22 ± 4.18), and their partners were around 31 years old (31.43 ± 4.57). Most of them were married (85.80%). On average, pregnant females had 14 years of education experience (14.96 ± 2.32), and their partners had about 15 years of education (15.19 ± 2.53). 3.55% of pregnant women and 47.93% of male partners were involved in tobacco consumption. Only four pregnant females had a history of depressive disorder (2.37%). Participants’ clinical characteristics, including the perceived general family functioning, childhood traumatic experience, quality of life, and social support, were all shown in Table 1.

**Table 1:** Characteristics of females and their partners (N = 169 dyads)

Item	Pregnant women	Partners
Age in years, mean (SD ^a^)	29.22 ± 4.18	31.43 ± 4.57
BMI ^b^ (kg/m^2^), mean (SD)	21.63 ± 3.17	23.78 ± 3.37
Educational years, mean (SD)	14.96 ± 2.32	15.19 ± 2.53
Married, n (%)	145 (85.80%)	145 (85.80%)
Employed, n (%)	121 (71.60%)	167 (98.82%)
History of drinking, n (%)	9 (5.33%)	91 (53.85%)
History of smoking, n (%)	6 (3.55%)	81 (47.93%)
Gestational days, mean (SD)	48.11 ± 12.03	-
Any history of depressive disorder, n (%)	4 (2.37%)	-
Any history of anxiety disorder, n (%)	1 (0.59%)	-
FAD-GF ^c^, mean (SD)	1.76 ± 0.44	1.84 ± 0.38
CTQ-SF ^d^, median (IQR ^e^)	40 (10)	-
Q-LES-Q-SF ^f^	52.71 ± 7.58	-
SSRS ^g^		
Total score, mean (SD)	38.08 ± 6.04	-
Objective support	10.57 ± 2.63	-
Subjective support	19.44 ± 3.37	-
Utilization of support	8.08 ± 1.81	-

^a^ SD, Standard Deviation; ^b^ BMI, Body Mass Index; ^c^ FAD-GF, Family Assessment Device-General Functioning; ^d^ CTQ-SF, Childhood Trauma Questionnaire-28 Item Short Form; ^e^ IQR, Interquartile Range; ^f^ Q-LES-Q-SF, Quality of Life Enjoyment and Satisfaction Questionnaire, Short Form; ^g^ SSRS, Social Support Rating Scale

### Anxious and depressive symptoms in females and their partners

As shown in Table 2, among pregnant women, 17.75% of them experienced clinically significant depressive symptoms (EPDS ≥ 10 points). Meanwhile, there were 11.83% of male partners experienced depressive symptoms (PHQ-9 ≥ 10 points). As the evaluation of the GAD-7 scale reported, 5.92% of pregnant females and 9.47% of partners had significant anxious symptoms (GAD-7 ≥ 10 points).

**Table 2:** Anxious and depressive symptoms in females and their partners

Item		Pregnant women	Partners
EPDS ^a^	total score, median (IQR ^b^)	6 (5)	-
	≥ 10 points, n (%)	30 (17.75%)	-
PHQ-9 ^c^	total score, median (IQR)	-	4 (6)
	≥ 10 points, n (%)	-	20 (11.83%)
GAD-7 ^d^	total score, median (IQR)	3 (4)	2 (6)
	≥ 10 points, n (%)	10 (5.92%)	16 (9.47%)

^a^ EPDS, Edinburgh Postnatal Depression Scale; ^b^ IQR, Interquartile Range; ^c^ PHQ-9, Patient Health Questionnaire-9; ^d^ GAD-7, Generalized Anxiety Disorder 7-Item

### Factors related to anxious and depressive symptoms and their effects

Factors related to mood symptoms in females and their partners were shown in Table 3. As we can see in females, age in years was negatively correlated with scores of EPDS, and a history of depressive disorder was positively correlated to scores of GAD-7 (r coefficient = − 0.17 and 0.16, respectively; both *P*_*r*_< 0.05). Meanwhile, scores of FAD-GF, Q-LES-Q-SF, and SSRS (total score, and subjective support) were all associated with scores of EPDS and GAD-7 (all *P*_*r*_< 0.01) in female participants. In partners, their depressive symptoms were correlated with educational years, marital status, history of smoking, and scores of FAD-GF (r coefficient = − 0.18, − 0.18, 0.16, and 0.33, respectively; all *P*_*r*_< 0.05). In addition, educational level and scores of FAD-GF were both related to their anxious symptoms (r coefficient = − 0.16 and 0.33, respectively; both *P*_*r*_< 0.05).

**Table 3:** Factors related to anxious and depressive symptoms in females and partners

Item	Pregnant women			Partners	
	EPDS ^e^	GAD-7 ^f^		PHQ-9 ^g^	GAD-7
Age in years ^c^	**−0.17** ^*^	−0.06		−0.06	−0.08
BMI ^c, h^	−0.05	−0.09		−0.06	−0.08
Educational years ^c^	−0.11	−0.07		**−0.18** ^*^	**−0.16** ^*^
Married ^c^	−0.09	−0.07		**−0.18** ^*^	−0.13
Employed ^c^	0.02	< 0.01		−0.03	0.004
History of drinking ^c^	0.06	−0.03		0.08	0.10
History of smoking ^c^	0.01	0.02		**0.16** ^*^	0.09
Gestational days ^c^	−0.05	−0.10		-	-
Any history of depressive disorder ^c^	0.10	**0.16** ^*^		-	-
Any history of anxiety disorder ^c^	0.13	0.13		-	-
FAD-GF ^d, I^	**0.47** ^***^	**0.41** ^***^		**0.33** ^***^	**0.33** ^***^
CTQ-SF ^d, j^	0.15	0.13		-	-
Q-LES-Q-SF ^d, k^	**−0.57** ^***^	**−0.49** ^***^		-	-
SSRS ^d, l^				-	-
Total score	**−0.29** ^***^	**−0.21** ^**^		-	-
Objective support	−0.15	−0.14		-	-
Subjective support	**−0.24** ^**^	**−0.21** ^**^		-	-
Utilization of support	**−0.25** ^**^	−0.11		-	-

^a^ Data are presented as r coefficients; ^b^ bold values indicate statistical significance; ^c^ Spearman correlation analysis; ^d^ Spearman partial correlation analysis; ^e^ EPDS, Edinburgh Postnatal Depression Scale; ^f^ GAD-7, Generalized Anxiety Disorder 7-Item; ^g^ PHQ-9, Patient Health Questionnaire-9; ^h^ BMI, body mass index; ^I^ FAD-GF, Family Assessment Device-General Functioning; ^j^ CTQ-SF, Childhood Trauma Questionnaire-28 Item Short Form; ^k^ Q-LES-Q-SF, Quality of Life Enjoyment and Satisfaction Questionnaire, Short Form; ^l^ SSRS, Social Support Rating Scale. ^*^P_*r*_< 0.05; ^**^P_*r*_< 0.01; ^***^P_*r*_< 0.001

As Table 4 shown, we included variables significant in correlation analyses and with *P* ≤ 0.1 in the univariate logistic regression analyses to the multivariate logistic regression analysis model to evaluate the effects of these risk factors on depressive and anxious symptoms. Finally, higher scores of FAD-GF (OR = 5.46 and 13.09, respectively; both *P* < 0.05) and lower scores of Q-LES-Q-SF (OR = 0.83 and 0.70, respectively; both *P* < 0.01) were significant risk factors for females’ depressive and anxious symptoms.

**Table 4:** Effects of risk factors on anxious and depressive symptoms in females

Factor	Depressive symptoms (Yes / No)						Anxious symptoms (Yes / No)				
	Univariate logistic regression			Multivariate logistic regression			Univariate logistic regression			Multivariate logistic regression	
	OR ^b^ (95% CI ^c^)	*P* value		OR (95% CI)	*P* value		OR (95% CI)	*P* value		OR (95% CI)	*P* value
Age in years	0.88 (0.79–0.97)	**0.01**		0.86 (0.74–0.99)	**0.04**		-	-		-	-
Any history of depressive disorder	-	-		-	-		5.78 (0.55–61.24)	0.15		-	-
FAD-GF ^d^	15.95 (5.11–49.80)	**< 0.01**		5.46 (1.47–20.36)	**0.01**		2.41 (0.06–91.26)	**< 0.01**		13.09 (1.51-113.16)	**0.02**
Q-LES-Q-SF ^e^	0.79 (0.73–0.86)	**< 0.01**		0.83 (0.76–0.91)	**< 0.01**		0.69 (0.58–0.83)	**< 0.01**		0.70 (0.57–0.87)	**< 0.01**
SSRS ^f^											
Total score	0.89 (0.83–0.96)	**< 0.01**		0.92 (0.71–1.20)	0.54		0.89 (0.80-1.00)	**0.05**		1.15 (0.84–1.58)	0.39
Subjective support	0.86 (0.76–0.97)	**0.02**		1.21 (0.83–1.78)	0.32		0.78 (0.63–0.97)	**0.02**		0.76 (0.45–1.29)	0.31
Utilization of support	0.74 (0.58–0.94)	**0.02**		0.93 (0.59–1.46)	0.74		-	-		-	-

^a^ Bold values indicate *P* ≤ 0.1 in univariate logistic regression and *P* **<** 0.05 in multivariate logistic regression; ^b^ OR, Odds Ratio; ^c^ CI, Confidence Interval; ^d^ FAD-GF, Family Assessment Device-General Functioning; ^e^ Q-LES-Q-SF, Quality of Life Enjoyment and Satisfaction Questionnaire, Short Form; ^f^ SSRS, Social Support Rating Scale

As Table 5 shown, in partners, the smoking history was significantly related to the risk of depressive symptoms (OR = 4.49, *P* = 0.01). Meanwhile, higher scores of FAD-GF were associated with the risk of depressive and anxious symptoms (OR 3.95 and 6.89, respectively; both *P* < 0.05).

**Table 5:** Effects of risk factors on anxious and depressive symptoms in partners

Factor	Depressive symptoms (Yes / No)						Anxious symptoms (Yes / No)				
	Univariate logistic regression			Multivariate logistic regression			Univariate logistic regression			Multivariate logistic regression	
	OR ^b^ (95% CI ^c^)	*P* value		OR (95% CI)	*P* value		OR (95% CI)	*P* value		OR (95% CI)	*P* value
Educational years	1.01 (0.84–1.22)	0.91		-	-		0.84 (0.69–1.02)	**0.08**		0.92 (0.74–1.13)	0.42
Married	0.32 (0.11–0.94)	**0.04**		0.45 (0.15–1.40)	0.17		-	-		-	-
History of smoking	5.17 (1.65–16.20)	**< 0.01**		4.49 (1.40–14.40)	**0.01**		-	-		-	-
FAD-GF ^d^	4.59 (1.33–15.81)	**0.02**		3.95 (1.06–14.67)	**0.04**		8.36 (2.05–34.09)	**< 0.01**		6.89 (1.58–29.97)	**0.01**

^a^ Bold values indicate *P* ≤ 0.1 in univariate logistic regression and *P* **<** 0.05 in multivariate logistic regression; ^b^ OR, Odds Ratio; ^c^ CI, Confidence Interval; ^d^ FAD-GF, Family Assessment Device-General Functioning

## Discussion

The current study noted that 17.75% of the first-trimester women experienced depressive symptoms, and 5.92% of them experienced anxious symptoms during the pandemic era of COVID-19 in China. A part of their partners also engaged in depressive (11.83%) and anxious symptoms (9.47%), which should not be overlooked. Furthermore, risk factors of mood symptoms such as family functioning, quality of life, and smoking history were stressed during early pregnancy.

### Prevalence of anxious and depressive symptoms in first-trimester females and their partners

Compared to previous studies during the COVID-19 pandemic, rates of perinatal anxious and depressive symptoms between countries differed significantly [32]. In some European countries using the same instruments for assessment, such as in Spain, the prevalence of maternal perinatal depression and anxiety was 47.2% and 33.3%, respectively [45], or in Sweden, 43.2% of pregnant women reported depression and 25.7% of them with anxiety [33]. In the Americas, the prevalence of clinically significant depressive and anxious symptoms was also relatively higher than in the current study [32]. Generally, people in countries with more confirmed COVID-19 cases and related deaths may have a higher risk for greater psychological distress. Meanwhile, different governmental restriction policies and measures were also related to outcomes of mental health during this period [46].

Compared results of the current study with research in different perinatal periods in China, Zhang L et al. reported a higher prevalence of depression in a third-trimester sample from Anhui Province (19.1%) before the COVID-19 pandemic [47] while Yu Y et al. applied a cluster random sampling method in urban areas of Hengyang City, Hunan Province and found a lower prevalence of depressive symptoms in late pregnancy (9.2%) [48]. The inconsistent results may be related to regional differences, different methods of sample acquisition, different assessment tools and threshold values. However, they validate differences in the prevalence of depressive symptoms in different trimesters.

Meanwhile, in the present study, the proportion of pregnant females with significant depressive symptoms (17.75%) was higher than those with significant anxious symptoms (5.92%), which was contrary to research before the COVID-19 pandemic in China. For example, a study enrolling first-trimester females from Chongqing, China, reported 15.04% of anxious symptoms compared to 5.19% with depressive symptoms [49]. The reason for the difference may be associated with the different effects of the pandemic of COVID-19 on perinatal mental health. According to a systematic review and meta-analysis exploring the effects of the pandemic on maternal and perinatal outcomes, an increase in maternal deaths, stillbirth, and depression are reported during the pandemic compared with the pre-pandemic [50], pointing that more clinical attention and interventions are necessary and should be given for prenatal depression.

Notably, the prevalence of male partners’ symptoms of anxiety was higher than that of pregnant females (9.47% vs. 5.92%) in this study. As previous studies suggested, expectant fathers are prone to anxiety [15]. Furthermore, men may bear more financial pressure in a family under the traditional Chinese culture. However, the COVID-19 pandemic has affected the economy, and increased employment and financial stress [18], which might result in significant anxiety among males [19]. The prevalence of their depressive symptoms in the present study (11.83%) was also significantly higher than data before the epidemic [20]. Therefore, mental health screening in early pregnancy for male partners should be intensified and necessary worldwide action should be performed to support pregnant families at times of crisis [51].

### Worse family functioning and quality of life linked to increased risk of anxious and depressive symptoms in first-trimester females

The increased risk of depressive and anxious symptoms was linked to poorer family functioning in the current study, which was supported by the research of Wu et al. reporting that pregnant women with moderate or severe family dysfunction have an increased likelihood of anxious symptoms [9]. According to the McMaster Model of Family Functioning (MMFF) theory [52], the basic function of the family is to provide conditions for the well-being of its members. Dysfunctional families affect individuals’ physical and mental health and are associated with the severity and course of depression and anxiety in members [53].

The exact reasons why worse family functioning was related to increased risk of mood symptoms in first-trimester females were unknown. We guessed that there might be two reasons. Firstly, previous studies revealed that adverse family events, marital strains, and spouses’ emotional problems are intense stressors related to depression [54, 55]. Poorer family functioning might act as a stressor leading to mood symptoms in pregnant women. Secondly, family functioning is a critical component of quality of life. Quality of life is also associated with anxiety [56] and depressive symptoms in pregnant women [57]. The association between family functioning and maternal mood symptoms may be mediated by the quality of life, which requires further investigation.

### Worse family functioning and history of smoking linked to increased risk of anxious and depressive symptoms in partners

Previous studies showed that smoking is significantly correlated with depression [58–60]. In the present study, a history of smoking was associated with an increased risk of depressive symptoms in partners, which emphasized the importance of screening for tobacco consumption among them in early pregnancy. Meanwhile, we also found an association between family functioning and partners’ risk of mood symptoms, which requires special attention.

To our knowledge, this is the first study to investigate the prevalence and risk factors of anxious and depressive symptoms among Chinese first-trimester females and their partners during the COVID-19 pandemic era. Results called for increased screening of mood symptoms in the risk groups with worse family functioning, lower quality of life, or a history of smoking.

## Limitations

(1) This study used self-assessment screening tools to assess anxious and depressive symptoms. A structured clinical interview was not used for diagnosis. (2) Family functioning was also self-assessed in the present study. The clinician-rated McMaster Clinical Rating Scale and the clinician-directed McMaster Structured Interview were not included. Subjective factors might influence the assessment of family functioning. (3) The Criterion Validity of each scale used in this study could not be explored because of the lack of the corresponding measurement instrument as a validity standard. (4) Data collection from partners was primarily performed online. Considering the relative complexity to finish the Q-LES-Q-SF, CTQ-SF, and SSRS scales, on-site instruction may be required. Therefore, we failed to assess the quality of life, childhood trauma history, and social support in partners, which needs to be explored in the future. (5) A randomized sampling method was not applied in this study, which may not be conducive to the generalization of the results to the overall population. (6) Previous studies found that family therapy based on the MMFF theory effectively could improve family functioning [61]. However, this study was observational and did not explore whether family therapy based on this theory could improve the emotional symptoms of pregnant females and their partners.

## Conclusion

Depressive and anxious symptoms are common in first-trimester females and their partners during the pandemic era of COVID-19 in China. 17.75% and 5.92% of first-trimester females had significant symptoms of depression and anxiety, respectively. In addition, 11.83% and 9.47% of male partners reported symptoms of depression and anxiety, respectively. The family functioning, quality of life, and smoking history increased the risk of mood symptoms. The findings in the current study emphasized the importance of early mental health screening for vulnerable families during the pandemic. It was also beneficial to promote updated guidance on prevention and interventions for pregnant females and their partners facing clinically significant symptoms of anxiety and depression.

## Data Availability

The datasets used and/or analyzed during the current study are available from the corresponding author upon reasonable request.

## References

[1] Giardinelli L, Innocenti A, Benni L, Stefanini MC, Lino G, Lunardi C, et al. Depression and anxiety in perinatal period: prevalence and risk factors in an italian sample. Arch Womens Ment Health. 2012 Feb;15(1):21–30. 10.1007/s00737-011-0249-8 22205237

[2] Kurki T. Depression and anxiety in early pregnancy and risk for preeclampsia. Obstet Gynecol. 2000 Apr;95(4):487–90. 10.1016/s0029-7844(99)00602-x 10725477

[3] Grant KA, McMahon C, Austin MP. Maternal anxiety during the transition to parenthood: A prospective study.Journal of Affective Disorders. 2008May;108(1–2):101–11. 10.1016/j.jad.2007.10.002 18001841

[4] Madigan S, Oatley H, Racine N, Fearon RMP, Schumacher L, Akbari E, et al. A Meta-analysis of maternal prenatal depression and anxiety on child Socioemotional Development. J Am Acad Child Adolesc Psychiatry. 2018 Sep;57(9):645–657e8. 10.1016/j.jaac.2018.06.012 30196868

[5] Wu YC, Chen CS, Chan YJ. The outbreak of COVID-19: an overview. J Chin Med Association. 2020 Mar;83(3):217–20. 10.1097/JCMA.0000000000000270 PMC7153464 32134861

[6] Farrell T, Reagu S, Mohan S, Elmidany R, Qaddoura F, Ahmed EE et al. The impact of the COVID-19 pandemic on the perinatal mental health of women.Journal of Perinatal Medicine. 2020 Nov26;48(9):971–6. 10.1515/jpm-2020-0415 32975206

[7] Lin W, Wu B, Chen B, Lai G, Huang S, Li S, et al. Sleep conditions associate with anxiety and depression symptoms among pregnant women during the epidemic of COVID-19 in Shenzhen. J Affect Disord. 2021 Feb;281:567–73. 10.1016/j.jad.2020.11.114 PMC7688420 33261931

[8] Al-Memar M, Vaulet T, Fourie H, Bobdiwala S, Farren J, Saso S, et al. First‐trimester intrauterine hematoma and pregnancy complications. Ultrasound Obstet Gynecol. 2020 Apr;55(4):536–45. 10.1002/uog.20861 31483898

[9] Wu F, Lin W, Liu P, Zhang M, Huang S, Chen C, et al. Prevalence and contributory factors of anxiety and depression among pregnant women in the post-pandemic era of COVID-19 in Shenzhen, China. J Affect Disord. 2021 Aug;291:243–51. 10.1016/j.jad.2021.05.014 PMC9754805 34051531

[10] Teixeira C, Figueiredo B, Conde A, Pacheco A, Costa R. Anxiety and depression during pregnancy in women and men. J Affect Disorders. 2009 Dec;119(1–3):142–8. 10.1016/j.jad.2009.03.005 19346001

[11] Shi YJ, Lu Z, Cao LL, Li Y (2018). Study on the correlations between anxiety and depression during pregnancy and personality characteristics,social support. Maternal and Child Health Care of China.

[12] Lee AM, Lam SK, Sze Mun Lau SM, Chong CSY, Chui HW, Fong DYT. Prevalence, Course, and risk factors for antenatal anxiety and depression. Volume 110. Obstetrics & Gynecology; 2007 Nov. pp. 1102–12. 5. 10.1097/01.AOG.0000287065.59491.70 17978126

[13] Bunevicius R, Kusminskas L, Bunevicius A, Nadisauskiene RJ, Jureniene K, Pop VJM. Psychosocial risk factors for depression during pregnancy. Acta Obstet Gynecol Scand. 2009 Jan;88(5):599–605. 10.1080/00016340902846049 19308810

[14] Racine N, Zumwalt K, McDonald S, Tough S, Madigan S. Perinatal depression: the role of maternal adverse childhood experiences and social support. J Affect Disord. 2020 Feb;263:576–81. 10.1016/j.jad.2019.11.030 31759669

[15] Field T, Diego M, Hernandez-Reif M, Figueiredo B, Deeds O, Contogeorgos J, et al. Prenatal paternal depression. Infant Behav Dev. 2006 Dec;29(4):579–83. 10.1016/j.infbeh.2006.07.010 PMC1769552 17138311

[16] Figueiredo B, Conde A. Anxiety and depression in women and men from early pregnancy to 3-months postpartum. Arch Womens Ment Health. 2011 Jun;14(3):247–55. 10.1007/s00737-011-0217-3 21479759

[17] Glasser S, Lerner-Geva L. Focus on fathers: paternal depression in the perinatal period. Perspect Public Health. 2019 Jul;139(4):195–8. 10.1177/1757913918790597 30044191

[18] Debata B, Patnaik P, Mishra A. COVID -19 pandemic! It’s impact on people, economy, and environment. J Public Affairs [Internet]. 2020 Sep 2 [cited 2022 Jul 26]; Available from: https://onlinelibrary.wiley.com/doi/10.1002/pa.2372

[19] Moore HE, Siriwardena AN, Gussy M, Hill B, Tanser F, Spaight R. Exploring the impact of the COVID-19 pandemic on male Mental Health Emergencies attended by Ambulances during the First National “Lockdown” in the East Midlands of the United Kingdom. Am J Mens Health. 2022 Mar;16(2):155798832210824. 10.1177/15579883221082428 PMC8902032 35246002

[20] Koh YW, Chui CY, Tang CSK, Lee AM (2014). The prevalence and risk factors of Paternal Depression from the Antenatal to the Postpartum Period and the Relationships between Antenatal and Postpartum Depression among fathers in Hong Kong. Depress Res Treat.

[21] Silverman ME, Smith L, Lichtenstein P, Reichenberg A, Sandin S. The association between body mass index and postpartum depression: a population-based study. J Affect Disord. 2018 Nov;240:193–8. 10.1016/j.jad.2018.07.063 30077160

[22] Cox JL, Holden JM, Sagovsky R. Detection of postnatal depression. Development of the 10-item Edinburgh postnatal depression scale. Br J Psychiatry. 1987 Jun;150:782–6. 10.1192/bjp.150.6.782 3651732

[23] Liang P, Wang Y, Shi S, Liu Y, Xiong R. Prevalence and factors associated with postpartum depression during the COVID-19 pandemic among women in Guangzhou, China: a cross-sectional study. BMC Psychiatry. 2020 Dec;20(1):557. 10.1186/s12888-020-02969-3 PMC7686811 33238927

[24] Wang Y, Guo X, Lau Y, Chan KS, Yin L, Chen J. Psychometric evaluation of the Mainland Chinese version of the Edinburgh postnatal depression scale. Int J Nurs Stud. 2009 Jun;46(6):813–23. 10.1016/j.ijnurstu.2009.01.010 19217107

[25] Kroenke K, Spitzer RL, Williams JB. The PHQ-9: validity of a brief depression severity measure. J Gen Intern Med. 2001 Sep;16(9):606–13. 10.1046/j.1525-1497.2001.016009606.x PMC1495268 11556941

[26] Sun XY, Li YX, Yu CQ, Li LM. [Reliability and validity of depression scales of chinese version: a systematic review]. Zhonghua Liu Xing Bing Xue Za Zhi. 2017 Jan;10(1):110–6. 10.3760/cma.j.issn.0254-6450.2017.01.021 28100388

[27] Levis B, Benedetti A, Thombs BD. Accuracy of Patient Health Questionnaire-9 (PHQ-9) for screening to detect major depression: individual participant data meta-analysis.BMJ. 2019 Apr 9;l1476. 10.1136/bmj.l1476 PMC6454318 30967483

[28] Spitzer RL, Kroenke K, Williams JBW, Löwe B. A brief measure for assessing generalized anxiety disorder: the GAD-7. Arch Intern Med. 2006 May;22(10):1092–7. 10.1001/archinte.166.10.1092 16717171

[29] Gong Y, Zhou H, Zhang Y, Zhu X, Wang X, Shen B, et al. Validation of the 7-item generalized anxiety disorder scale (GAD-7) as a screening tool for anxiety among pregnant chinese women. J Affect Disord. 2021 Mar;282:98–103. 10.1016/j.jad.2020.12.129 33401129

[30] Shangguan F, Wang R, Quan X, Zhou C, Zhang C, Qian W, et al. Association of stress-related factors with anxiety among chinese pregnant participants in an Online Crisis intervention during COVID-19 epidemic. Front Psychol. 2021 Apr;30:12:633765. 10.3389/fpsyg.2021.633765 PMC8119994 33995188

[31] Williams N. The GAD-7 questionnaire. Occupational Medicine. 2014 Apr 1;64(3):224–224.

[32] Mateus V, Cruz S, Costa R, Mesquita A, Christoforou A, Wilson CA, et al. Rates of depressive and anxiety symptoms in the perinatal period during the COVID-19 pandemic: comparisons between countries and with pre-pandemic data. J Affect Disord. 2022 Nov;316:245–53. 10.1016/j.jad.2022.08.017 PMC9365708 35964769

[33] Ho-Fung C, Andersson E, Hsuan-Ying H, Acharya G, Schwank S. Self-reported mental health status of pregnant women in Sweden during the COVID-19 pandemic: a cross-sectional survey. BMC Pregnancy Childbirth. 2022 Dec;22(1):260. 10.1186/s12884-022-04553-x PMC8960205 35351030

[34] Green SM, Inness B, Furtado M, McCabe RE, Frey BN. Evaluation of an augmented cognitive behavioural Group Therapy for Perinatal generalized anxiety disorder (GAD) during the COVID-19 pandemic. JCM. 2021 Dec;31(1):209. 10.3390/jcm11010209 PMC8745906 35011950

[35] Epstein NB, Baldwin LM, Bishop DS. THE McMASTER FAMILY ASSESSMENT DEVICE*. J Marital Fam Ther. 1983 Apr;9(2):171–80.

[36] Shek DTL. Assessment of Family Functioning in Chinese Adolescents: The Chinese Version of the Family Assessment Device. Research on Social Work Practice. 2002 Jul;12(4):502–24.

[37] Shek DTL. The General Functioning Scale of the Family Assessment device: does it work with chinese adolescents? J Clin Psychol. 2001 Dec;57(12):1503–16. 10.1002/jclp.1113 11745592

[38] Jean E, John N, Wilma H, Richard B (1993). Quality of life enjoyment and satisfaction questionnaire: a new measure. Psychopharmacol Bull.

[39] Lee YT, Liu SI, Huang HC, Sun FJ, Huang CR, Yeung A. Validity and reliability of the chinese version of the short form of quality of life enjoyment and satisfaction questionnaire (Q-LES-Q-SF). Qual Life Res. 2014 Apr;23(3):907–16. 10.1007/s11136-013-0528-0 24062242

[40] Bernstein DP, Stein JA, Newcomb MD, Walker E, Pogge D, Ahluvalia T, et al. Development and validation of a brief screening version of the Childhood Trauma Questionnaire. Child Abuse Negl. 2003 Feb;27(2):169–90. 10.1016/s0145-2134(02)00541-0 12615092

[41] He J, Zhong X, Gao Y, Xiong G, Yao S. Psychometric properties of the chinese version of the Childhood Trauma Questionnaire-Short Form (CTQ-SF) among undergraduates and depressive patients. Child Abuse Negl. 2019 May;91:102–8. 10.1016/j.chiabu.2019.03.009 30856597

[42] Xiao SY (1994). The theoretical basis and research application of social support rating scale. J Clin Psychiatry.

[43] Fu C, Ren Y, Wang G, Shi X, Cao F. Fear of future workplace violence and its influencing factors among nurses in Shandong, China: a cross-sectional study. BMC Nurs. 2021 Dec;20(1):123. 10.1186/s12912-021-00644-w PMC8262060 34233678

[44] Huang Y, Liu Y, Wang Y, Liu D. Family function fully mediates the relationship between social support and perinatal depression in rural Southwest China. BMC Psychiatry. 2021 Dec;21(1):151. 10.1186/s12888-021-03155-9 PMC7953569 33711987

[45] Motrico E, Domínguez-Salas S, Rodríguez-Domínguez C, Gómez-Gómez I, Rodríguez-Muñoz MF, Gómez-Baya D. The Impact of the COVID-19 Pandemic on Perinatal Depression and Anxiety: A Large Cross-sectional Study in Spain. Psicothema. 2022 May;(34.2):200–8. 10.7334/psicothema2021.380 35485532

[46] Lee Y, Lui LMW, Chen-Li D, Liao Y, Mansur RB, Brietzke E, et al. Government response moderates the mental health impact of COVID-19: a systematic review and meta-analysis of depression outcomes across countries. J Affect Disord. 2021 Jul;290:364–77. 10.1016/j.jad.2021.04.050 PMC8159271 34052584

[47] Zhang L, Wang L, Cui S, Yuan Q, Huang C, Zhou X. Prenatal depression in women in the third trimester: prevalence, predictive factors, and Relationship with maternal-fetal attachment. Front Public Health. 2021 Jan;26:8:602005. 10.3389/fpubh.2020.602005 PMC7870992 33575242

[48] Yu Y, Zhu X, Xu H, Hu Z, Zhou W, Zheng B, et al. Prevalence of depression symptoms and its influencing factors among pregnant women in late pregnancy in urban areas of Hengyang City, Hunan Province, China: a cross-sectional study. BMJ Open. 2020 Sep;10(9):e038511. 10.1136/bmjopen-2020-038511 PMC7467533 32873680

[49] Tang X, Lu Z, Hu D, Zhong X. Influencing factors for prenatal stress, anxiety and depression in early pregnancy among women in Chongqing, China. J Affect Disord. 2019 Jun;253:292–302. 10.1016/j.jad.2019.05.003 31077972

[50] Chmielewska B, Barratt I, Townsend R, Kalafat E, van der Meulen J, Gurol-Urganci I, et al. Effects of the COVID-19 pandemic on maternal and perinatal outcomes: a systematic review and meta-analysis. The Lancet Global Health. 2021 Jun;9(6):e759–72. 10.1016/S2214-109X(21)00079-6 PMC8012052 33811827

[51] Brown S. Perinatal mental health and the COVID -19 pandemic. World Psychiatry. 2020 Oct;19(3):333–4. 10.1002/wps.20779 PMC7491632 32931116

[52] Miller IW, Ryan CE, Keitner GI, Bishop DS, Epstein NB. The McMaster Approach to families: theory, assessment, treatment and research. J Family Therapy. 2000 May;22(2):168–89.

[53] Song J, Chen H, Liang T. Family functioning and 1-year prognosis of first-episode major depressive disorder. Psychiatry Res. 2019 Mar;273:192–6. 10.1016/j.psychres.2019.01.021 30654304

[54] Phelan J, Schwartz JE, Bromet EJ, Dew MA, Parkinson DK, Schulberg HC, et al. Work stress, family stress and depression in professional and managerial employees. Psychol Med. 1991 Nov;21(4):999–1012. 10.1017/s0033291700029998 1780412

[55] Sheeber L, Hops H, Davis B (2001). Family processes in adolescent depression. Clin Child Fam Psychol Rev.

[56] Wang J, Chen Y, Tan C, Zhao X. Family functioning, social support, and quality of life for patients with anxiety disorder. Int J Soc Psychiatry. 2016 Feb;62(1):5–11. 10.1177/0020764015584649 25964447

[57] Nicholson WK, Setse R, Hill-Briggs F, Cooper LA, Strobino D, Powe NR. Depressive Symptoms and Health-Related Quality of Life in Early Pregnancy: Obstetrics & Gynecology. 2006 Apr;107(4):798–806. 10.1097/01.AOG.0000204190.96352.05 16582115

[58] Boden JM, Fergusson DM, Horwood LJ. Cigarette smoking and depression: tests of causal linkages using a longitudinal birth cohort. Br J Psychiatry. 2010 Jun;196(6):440–6. 10.1192/bjp.bp.109.065912 20513853

[59] Paperwalla KN, Levin TT, Weiner J, Saravay SM. Smoking and depression. Med Clin North Am. 2004 Nov;88(6):1483–94. 10.1016/j.mcna.2004.06.007 15464109

[60] Wiesbeck GA, Kuhl HC, Yaldizli Ö, Wurst FM (2008). Tobacco Smoking and Depression – results from the WHO/ISBRA Study. Neuropsychobiology.

[61] Pourmovahed Z, Yassini Ardekani SM, Mazloomy Mahmoodabad SS, Zareei Mahmoodabadi H. Implementation of the McMaster Model in Family Therapy: Effects on Family Function in Married Couples. IJPS [Internet]. 2021 Feb 12 [cited 2022 Jul 30]; Available from: https://ec2-18-184-16-47.eu-central-1.compute.amazonaws.com/index.php/IJPS/article/view/5380 10.18502/ijps.v16i1.5380 PMC8140298 34054984

